# The Potential Mechanism of Cancer Patients Appearing More Vulnerable to SARS-CoV-2 and Poor Outcomes: A Pan-Cancer Bioinformatics Analysis

**DOI:** 10.3389/fimmu.2021.804387

**Published:** 2022-01-10

**Authors:** Xinwei Huang, Huazheng Liang, Hong Zhang, Li Tian, Peilin Cong, Tingmei Wu, Qian Zhang, Xiaofei Gao, Wanrong Li, Aiwen Chen, Yuxin Zhang, Qianyu Dong, Hanxi Wan, Mengfan He, Danqing Dai, Zhen Li, Lize Xiong

**Affiliations:** Translational Research Institute of Brain and Brain-Like Intelligence, Shanghai Fourth People’s Hospital, School of Medicine, Tongji University, Shanghai, China

**Keywords:** COVID-19, cancer, rs10774671 (OAS1), SARS-CoV-2, ACE2

## Abstract

To explore the potential mechanism of cancer patients appearing more vulnerable to SARS-CoV-2 infection and poor COVID-19 outcomes, we conducted an integrative bioinformatics analysis for SARS-CoV-2-required genes and host genes and variants related to SARS-CoV-2 susceptibility and COVID-19 severity. BLCA, HNSC, KIRC, KIRP, LGG, PCPG, PRAD, TGCT, and THCA patients carrying rs10774671-A (OAS1) genotype may be more likely to have poor COVID-19 outcomes relative to those who carry rs10774671-G, because individuals carrying rs10774671-A will have lower expression of OAS1, which serves as a protective factor against SARS-CoV-2 processes and poor COVID-19 outcomes. SARS-CoV-2-required genes were correlated with TME, immune infiltration, overall survival, and anti-cancer drug sensitivity. CHOL patients may have a higher risk of SARS-CoV-2 infection than healthy subjects. SARS-CoV-2-induced ACE2 and NPC1 elevation may have a negative influence on the immune responses of LUSC and CD8+T infiltration of LUAD, and negatively affect the sensitivity of anti-lung cancer drugs. LUSC and LUAD patients may have a varying degree of adverse outcomes if they are infected with SARS-CoV-2. miR-760 may target and inhibit ACE2 expression. Cancer patients appearing vulnerable to SARS-CoV-2 infection and having poor COVID-19 outcomes may be partly due to host genetic factors and dysregulation of SARS-CoV-2-required genes. OAS1, ACE2, and miR-760 could serve as the treatment and intervention targets for SARS-CoV-2.

## Introduction

**Figure 1:**
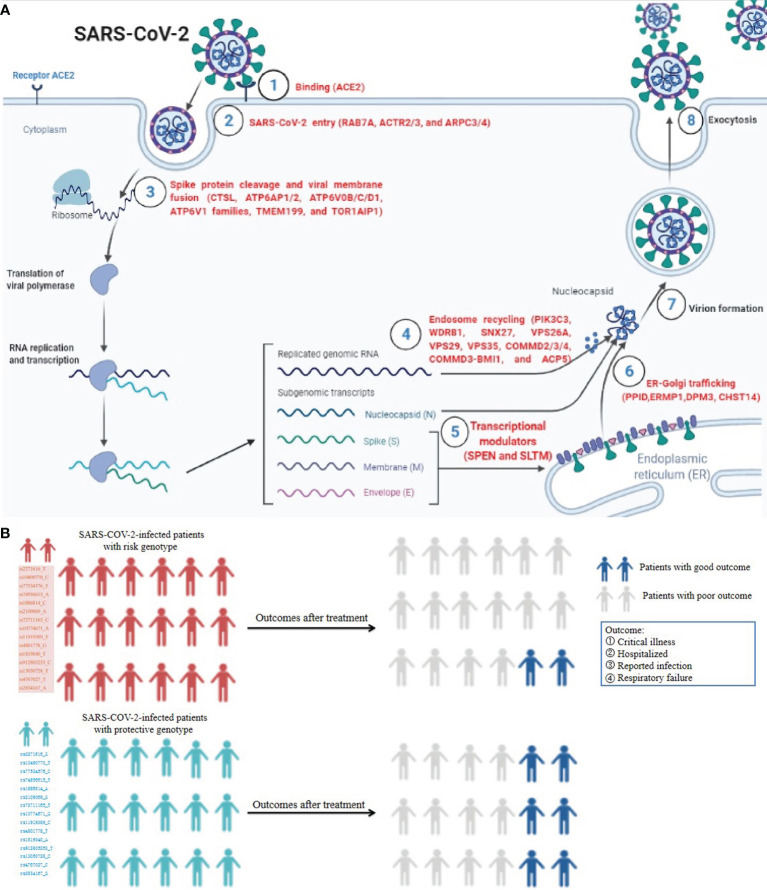
The roles of SARS-CoV-2-required genes in SARS-CoV-2 infection processes and influence of host genetic factor in COVID-19 outcomes. **(A)** The vital host genes required for SARS-CoV-2 infection processes containing initial binding, endosomal entry, spike protein cleavage, and viral membrane fusion, endosome recycling, ER-Golgi trafficking, and transcriptional modulators. **(B)** SARS-COV-2-infected patients with risk genotypes appear more vulnerable to SARS-COV-2 infection and poor outcome, while those carrying protective genotypes appear more vulnerable to lower SARS-COV-2 possibility and good outcome^5-9^. This figure is drawn on the Biorender website at https://biorender.com/.

As of 24 August 2021, 2019 novel coronavirus (COVID-19), which is caused by severe acute respiratory syndrome coronavirus 2 (SARS-CoV-2), has infected more than 200 million patients, including 4.2 million deaths (https://covid19.who.int/). Recently, some vital host genes required for SARS-CoV-2 infection processes containing initial binding (ACE2), endosomal entry (RAB7A, ACTR2/3, and ARPC3/4), spike protein cleavage, and viral membrane fusion (CTSL, TMPRSS2, TMEM199, ATP6AP1/2, ATP6V0B/C/D1, ATP6V1 families, and TOR1AIP1), endosome recycling (PIK3C3, WDR81, SNX27, VPS26A, VPS29, VPS35, COMMD2/3/4, COMMD3-BMI1, and ACP5), ER-Golgi trafficking (PPID, ERMP1, DPM3, and CHST14), and transcriptional modulators (SPEN and SLTM) were identified by Daniloski et al. (1) and Hoffmann et al. (2) using a genome-scale CRISPR loss-of-function screen or protease inhibitor in human cell lines (**Figure 1A**). By using single-cell transcriptomics, RNA interference knockdown, and small-molecule inhibitors, the loss of endosomal entry pathway genes ATP6AP1, ATP6V1A, CCDC22, NPC1, PIK3C3, and RAB7A was validated to result in increased cellular cholesterol, which can block SARS-CoV-2 infection (1–3). Severe acute respiratory syndrome coronavirus (SARS-CoV) and SARS-CoV-2 share 79.5% homologous sequences, and both viruses use similar host genes as receptors to enter human body cells (2). Kong and colleagues (4) indicated that normal lung and lung cancer cell lines infected with SARS-CoV can elevate ACE2 expression, maintaining a high level of expression at 1 and 2 days. Notably, several research teams have demonstrated that SARS-COV-2-infected patients with risk genotypes appear more vulnerable to SARS-COV-2 infection and poor outcomes, while those who carry protective genotypes appear more vulnerable to lower SARS-COV-2 possibility and good outcomes (5–9), indicating host-specific genetic factors play an important role in SARS-CoV-2 susceptibility and COVID-19 outcomes (**Figure 1B**). These findings provide new insights into the mechanisms of pathogenesis of SARS-CoV-2 susceptibility and poor outcomes.

Risk factors for severe events and deaths from SARS-CoV-2 infection include older age, smoking, and medical comorbidities, which are common in cancer patients. Four studies analyzing cancer patients with SARS-CoV-2 infection revealed that they appear more vulnerable to SARS-CoV-2 and show more deteriorating conditions and poor outcomes compared with non-cancer patients (10–13). Bernard et al. (10) and Dai et al. (11) indicated that patients with different cancer types (especially lung cancer and hematological cancer) and late metastatic stage have the highest frequency of severe events. The possible reasons for this may be attributed to cancer-related immunosuppression, known complications, and immunotherapy treatment (11, 13). However, the exact mechanisms remain unclear. Given a large number of cancer patients and the continuing spread of SARS-CoV-2, exploring this molecular mechanism could contribute to the treatment of cancer patients infected with SARS-CoV-2.

This study explores the potential mechanism for cancer patients appearing vulnerable to SARS-CoV-2 and poor outcomes *via* integrative bioinformatics analyses for SARS-CoV-2-required genes (ACE2, TMPRSS2, ATP6AP1, ATP6V1A, CCDC22, NPC1, PIK3C3, and RAB7A), host genes, and variants related to SARS-CoV-2 susceptibility and COVID-19 severity.

## Materials and Methods

### Data Download and Processing

RNA-seq and clinical data of 33 cancer types, and pan-cancer immune signature scores, stemness score, and stemness score data were downloaded from The Cancer Genome Atlas (TCGA) database *via* UCSC Xena (https://xena.ucsc.edu/). Drug susceptibility data including DTP NCI-60 and RNA-seq were obtained from the CellMiner database (https://discover.nci.nih.gov/cellminer/). In addition, RNA-seq datasets (GSE163959 and GSE147507) of human nasal turbinate, lung tissues, A549 cells, and primary human bronchial epithelial cells (NHBEs) with or without SARS-CoV-2 infection were downloaded from Gene Expression Omnibus (GEO) database (https://www.ncbi.nlm.nih.gov/geo/).

### Expression Analysis of SARS-CoV-2-Required Genes and Host Susceptibility Genes in Human Tissues and Cells After SARS-CoV-2 Infection

Package edgeR was used to normalize GSE163959 and GSE147507 raw count datasets. The t-test was utilized to compare the expression of SARS-CoV-2-required genes between control and SARS-CoV-2 infected samples. Package pheatmap was utilized to show their expression status. The same analysis was also performed for host-specific genes associated with COVID-19 susceptibility and severity. *P* < 0.05 was considered statistically significant.

### Expression Quantitative Trait Locus Analysis for Host Genes and Variants Related to COVID-19 Susceptibility and Severity

PancanQTL web platform was used to comprehensively evaluate the effect of variants related to COVID-19 susceptibility and severity on local gene expression (cis-eQTLs) in 33 cancer types. This platform included the expression and genotype data of 9,196 tumor samples and 5,606,570 cis-eQTL-gene pairs in 33 cancer types from TCGA (14). We then assessed the expression status of host genes related to COVID-19 severity in multiple organs and tumor tissues *via* The Human Protein Atlas database (https://www.proteinatlas.org/).

### Evaluating Expression Profiles of SARS-CoV-2-Required Genes Across Human Tissues

To identify the expression profiles of eight SARS-CoV-2-required genes across human tissues, we examined their expression across 21 tissue types using 4,790 RNA-seq datasets from the Genotype-Tissue Expression (GTEx) v8 database (https://www.gtexportal.org/home/datasets).

### Differential Expression Analysis for SARS-CoV-2-Required Genes Across 33 Cancer Types

Differential expression analysis for SARS-CoV-2-required genes was performed across 33 cancer types by wilcox.test function. R package pheatmap was used to visualize their differential expression status between cancer samples and non-cancer samples. *P* < 0.05 was considered statistically significant.

### Identification of SARS-CoV-2-Required Genes Associated With the Stage and Prognosis of Cancer Patient

Differential expression analysis between SARS-CoV-2-required genes and stage types in pan-cancers was performed using Gene Set Cancer Analysis (GSCA) database (http://bioinfo.life.hust.edu.cn/GSCA/#/expression) (15) and GEPIA 2 database (http://gepia2.cancer-pku.cn/#index) (16). Moreover, we used R packages survival, survminer, and reshape2 to explore the association between the expression of SARS-CoV-2-required genes and the prognosis of cancer patients. Firstly, based on the survival data, Kaplan–Meier curve was utilized to analyze the overall survival according to the high and low expression values of the gene. We then conducted the univariate Cox regression analysis for the relationship between the overall survival and expressions of SARS-CoV-2-required genes. *P* < 0.05 was considered statistically significant.

### Tumor Microenvironment Analysis

Kruskal.test and R packages ggplot2, limma, and reshape2 were used to test the association between immune subtypes and the expressions of these genes according to the immune landscape of cancers (17). The correlation between SARS-CoV-2-required gene expression and tumor microenvironment (TME) was analyzed with Spearman correlation and R packages estimate, limma, and corrplot, according to the ESTIMATE immune, stromal, and estimate scores, which can analyze the infiltration levels of both stromal and immune cells in cancers (18). Furthermore, cancer stem cell-like properties of each patient were obtained from stemness scores based on transcriptomic mRNA (RNAss) and epigenetic DNA methylation (DNAss). The association of stemness scores with SARS-CoV-2-required genes was assessed by spearman analysis.

### Immune Infiltration Analysis

GSCA database (15) was used to conduct immune infiltration analysis for SARS-CoV-2-required genes. Additionally, differential expression analysis for interested immune cell types between tumor and adjacent normal tissues was performed in the ImmuCellAI database (http://bioinfo.life.hust.edu.cn/ImmuCellAI/#!/resource) using wilcoxon test (19). Survival analysis was conducted to compare survival curves between high and low immune cell abundance in one cancer by multivariable Cox proportional hazard model. Covariates contained immune cell infiltration and clinical factors (tumor stages, age, and gender). *P* value of the log-rank test as shown in each plot was used to compare the survival curves of the two groups. Kaplan-Meier plot for immune cell infiltration was drawn to visualize the survival difference.

### Assessment of Association Between SARS-CoV-2-Required/Susceptibility Genes and Cancer-Related Genes

To explore the association between key SARS-CoV-2-required/susceptibility genes and estimate their influence in cancer-related genes affecting the prognosis of cancers, we performed a survival analysis for SARS-CoV-2-required/susceptibility genes in lung cancer *via* the GEPIA2 database. Based on TCGA lung cancer tissues, we evaluated their correlation using spearman correlation analysis. In addition, we performed a differentially expressed gene analysis between lung cancer tissues and normal tissues *via* the limma package of R soft. |log2 (fold change)| > 2 and *P* value < 0.05 after being adjusted by false discovery rate were applied as the cutoff for differential gene expression screening. We then assessed their correlation with the prognosis of lung cancer by the GSCA database.

### Drug Sensitivity Analysis

To identify the relationship between drug sensitivity and SARS-CoV-2-required genes, we evaluated the correlation between the expression of each SARS-CoV-2-required gene and z-score for cell sensitivity data (GI50) by spearman correlation analysis based on DTP NCI-60 and RNA-seq data obtained from the CellMiner database (20). In addition, a similar analysis was also performed *via* the GSCA database based on Genomics of Drug Sensitivity in Cancer (GDSC) and Cancer Therapeutics Response Portal (CTRP). |Cor| > 0.20 and *P* < 0.01 were considered as statistically significant.

### Prediction and Analysis of Upstream MicroRNAs of SARS-CoV-2-Required Genes

Upstream binding microRNAs of SARS-CoV-2-required genes were predicted based on seven prediction programs, containing RNA22, miRmap, PicTar, microT, PITA, miRanda, and TargetScan in starBase 3.0 database (http://starbase.sysu.edu.cn/), which mainly focus on miRNA-target interactions (21). The predicted microRNAs were obtained according to their appearance in one or more programs. StarBase 3.0 then was used to analyze the correlation of SARS-CoV-2-required genes with microRNAs, and assess the expression status of microRNAs in pan-cancer and normal control tissues. Additionally, survival analysis of microRNAs was also performed.

### Statistical Analysis

All statistical analyses were based on R soft version 4.10 and attached packages. Wilcox test was utilized to determine differentially expressed SARS-CoV-2-required genes between normal and tumor samples. Spearman correlation analysis was utilized to assess the correlation between two variables. Log-rank tests and Kaplan-Meier curves were utilized to evaluate the relationship between gene expression and overall survival. *P* < 0.05 was considered statistically significant.

## Results

### OAS1 May Serve as a Protective Factor Against SARS-CoV-2 Infection and Poor COVID-19 Outcomes

**Figure 2:**
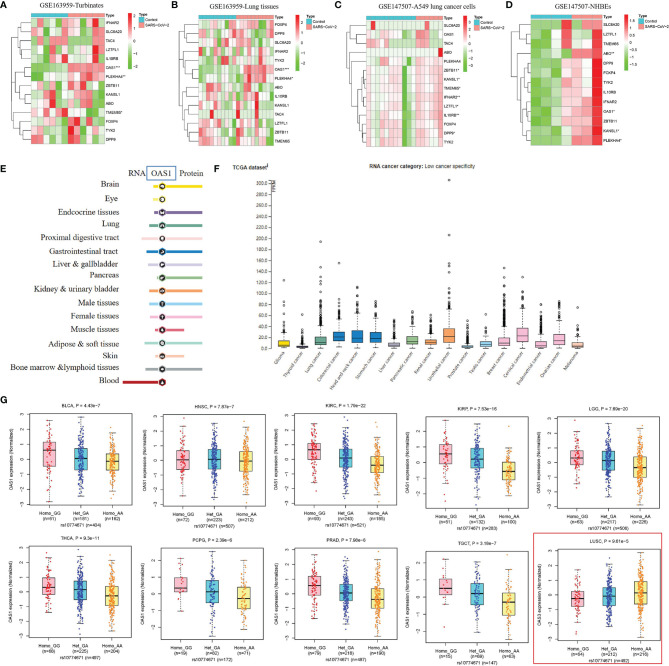
The role of genetic factors in SARS-CoV-2 susceptibility and COVID-19 outcomes. **(A–D)** The response of host genes related to SARS-CoV-2 susceptibility and COVID-19 outcomes in human tissues and cells infected with SARS-CoV-2. **(E, F)** Expression status of OAS1 in human organs and tumor tissues. This analysis was performed in The Human Protein Atlas (HPA) database (https://www.proteinatlas.org/). **(G)** The association of rs10774671-A (OAS1) with OAS1 expression in BLCA, HNSC, KIRC, KIRP, LGG, PCPG, PRAD, TGCT, and THCA tissues.

COVID-19 severity-related genes (SLC6A20, LZTFL1, FOXP4, TMEM65, ABO, OAS1, TAC4, DPP9, TYK2, ZBTB11, IL10RB, KANSL1, PLEKHA4, and IFNAR2) and single nucleotide polymorphisms (SNPs) identified by genome wide association studies were summarized in **Table S1** (5–9). To explore the role of genes related to COVID-19 severity in the process of SARS-CoV-2, we performed a differential expression analysis for these genes in human nasal turbinate, lung tissues, A549 cells (non-small cell lung cancer), and normal human bronchial epithelial cells (NHBEs) with/without SARS-CoV-2 infection. As shown in **Figures 2A–D** and **Table S2**, OAS1 and PLEKHA4 expressions were significantly elevated in turbinate, lung tissues, and NHBEs infected with SARS-CoV-2 compared to the control cells. In up to 14,134 cases and 1.2 million controls, Zhou et al. found that higher plasma OAS1 protein level is related to reduced susceptibility (OR = 0.78, *P* = 8 × 10-6), hospitalization (OR = 0.61, *P* = 8 × 10-8), and COVID-19 death or ventilation (OR = 0.54, *P* = 7 × 10-8) (9). We further explored whether OAS1 is specifically or widely expressed in organs and tumor tissues *via* The Human Protein Atlas (HPA) database (https://www.proteinatlas.org/). We found that OAS1 was broadly expressed in different human organs and tumor tissues, with low organ and cancer specificity (**Figures 2E, F**). These findings suggest that OAS1 may serve as a protective factor against SARS-CoV-2 progress and poor COVID-19 outcomes in the wide organs and tissues.

### Cancer Patients Carrying rs10774671-A (OAS1) Genotype May Appear Vulnerable to Poor COVID-19 Outcomes

COVID-19 severity-related SNPs were summarized in **Table S1**. Rs2271616-T (SLC6A20), rs10490770-C, rs11385942-GA (LZTFL1), rs1886814-C (FOXP4), rs72711165-C (TMEM65), rs505922-C (ABO), rs10774671-A (OAS1), rs77534576-T (TAC4), rs2109069-A (DPP9), and rs74956615-A (TYK2) was reported to increase the risk of SARS-CoV-2 susceptibility and poor COVID-19 outcomes such as critical illness, hospitalization, and respiratory failure, while rs11919389-C (ZBTB11), rs912805253-T (ABO), rs2834167-G (IL10RB), rs4767027-C (OAS1), rs1819040-A (KANSL1), rs4801778-T (PLEKHA4), and rs13050728-C (IFNAR2) decrease these risks (5–9). To further explore the effect of these genotypes on expressions of potential key genes, we performed an expression quantitative trait locus (cis-eQTL) analysis for these variants in 33 cancer types. As summarized in **Table S3**, rs4801778-T (PLEKHA4) was positively associated with TULP2 expression in LUAD (β = 0.26, *P*= 9.57E-05), while negatively associated with HSD17B14 expression in PAAD and PRAD (-0.37 < β < -0.36, 1.20E-08 < *P* < 8.36E-06). Rs11919389-C (ZBTB11) showed a positive relationship with LOC285359 and LOC100009676 expressions in GBM, LGG, PRAD, TGCT, or THCA (0.20 < β < 0.42, 3.78E-26 < *P* < 3.04E-05) and had a negative relationship with ZBTB11 and SENP7 expressions in LGG or OV (-0.24 < β < -0.14, 7.65E-05 < *P* < 9.95E-05). Rs13050728-C (IFNAR2) was positively linked to IFNAR2 expression in BRCA, LGG, and THCA (0.13 < β < 0.19, 5.58E-10 < *P* < 7.34E-05) and negatively associated with IL10RB expression in LGG (β = -0.16, *P* = 1.97E-06). In particular, rs10774671-A (OAS1) was positively related to OAS3 expression in LUSC (β = 0.20, *P* = 9.61E-05) and showed a consistent negative association with OAS1 expression in BLCA, HNSC, KIRC, KIRP, LGG, PCPG, PRAD, TGCT, and THCA (-0.57 < β < -0.23, 1.79E-22 < *P* < 2.39E-06) (**Figure 2G**). The above findings indicate that BLCA, HNSC, KIRC, KIRP, LGG, PCPG, PRAD, TGCT, and THCA patients carrying rs10774671-A (OAS1) genotype may be more likely to have poor COVID-19 outcomes relative to those carrying rs10774671-G because individuals carrying rs10774671-A will have lower expression of OAS1, which serves as a protective factor against SARS-CoV-2 infection and poor COVID-19 outcomes.

### SARS-CoV-2 Affects Expression Levels of SARS-CoV-2-Required Genes

**Figure 3:**
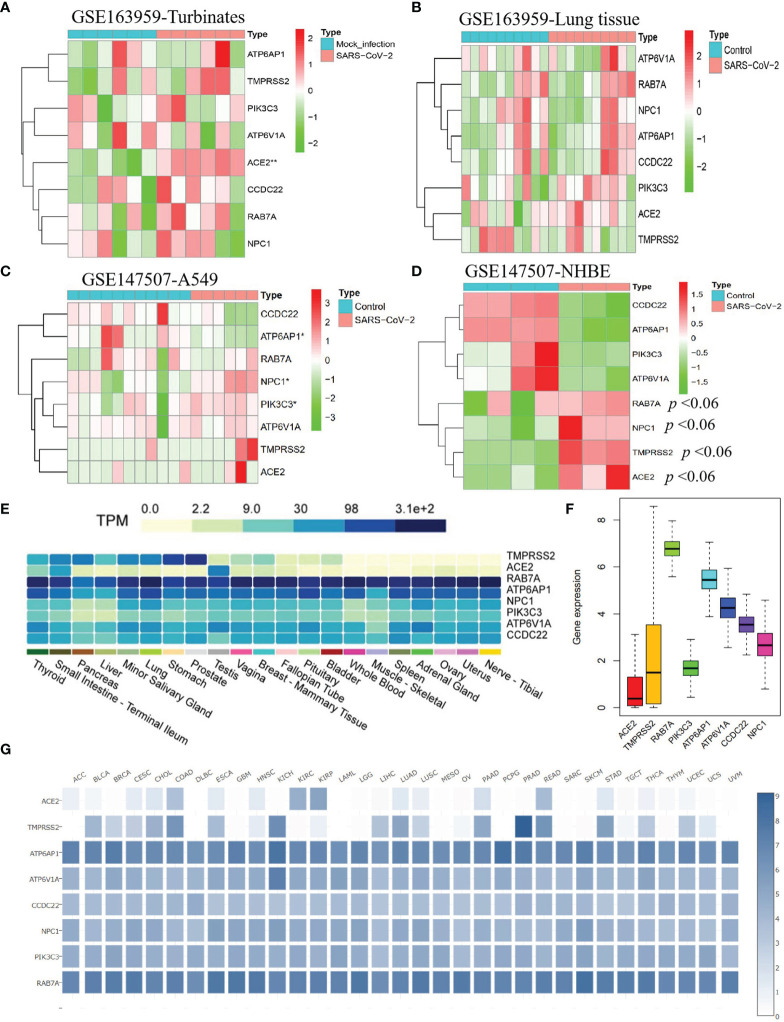
The response of SARS-CoV-2-required genes in human cells and tissues infected with SARS-CoV-2 and their expression status in human tissues and corresponding tumor tissues. The expression profiles of eight SARS-CoV-2-required genes in human nasal turbinate **(A)** and lung tissues **(B)**, A549 cells **(C)**, and primary human bronchial epithelial cells (NHBEs) **(D)** with/without SARS-CoV-2 infection. **(E)** Expression profiles of SARS-CoV-2-required genes in 21 human tissues. **(F)** Mean expression value of SARS-CoV-2-required genes in cancers. **(G)** Expression profiles of SARS-CoV-2-required genes across 33 cancer types.

Kong and colleagues found that SARS-CoV can obviously increase ACE2 and TMPRSS2 expression levels in Calu-3 cells during 24-48 hours compared with that at 12 hours (4), indicating this kind of virus may elevate the expression of SARS-CoV-2-required genes in human tissues or cells. To explore the influence of SARS-CoV-2 infection in SARS-CoV-2-required genes, we performed a differential expression analysis for eight SARS-CoV-2-required genes in human nasal turbinates and lung tissues, A549 cells, and NHBEs with/without SARS-CoV-2 infection. We found that ACE2 is significantly elevated in human nasal turbinate infected with SARS-CoV-2 compared with mock infected turbinate (*P* = 0.002). Furthermore, the expressions of ATP6AP1, NPC1, and PIK3C3 in A549 cells were significantly influenced by SARS-CoV-2 infection compared with the control group. In addition, NHBEs infected with SARS-CoV-2 also showed an obviously increased expression of ACE2, TMPRSS2, NPC1, and RAB7A compared to that in control NHBEs (*P* < 0.06) (**Figures 3A–D**). These results indicate that SARS-CoV-2 can affect the expression levels of SARS-CoV-2-required genes in human normal tissues or cells and lung tumor cells.

### Identification of SARS-CoV-2-Required Gene Expression in Human Tissues

SARS-CoV-2 was reported to invade various tissues such as the lung, nerve, adrenal, esophagus, thymus, pancreas, breast, skin cervix, and lymph node (22), with different susceptibility across these tissues (22, 23). In this study, we analyzed the expression profiles of SARS-CoV-2-required genes in normal tissue types and explored whether this might influence the susceptibility of the corresponding tissue tumor to SARS-CoV-2. Using 4,790 RNA-seq datasets from the Genotype-Tissue Expression (GTEx) v8 database, we evaluated their expression across 21 tissue types. We found that ACE2 and TMPRSS2 had an obvious expression difference between human tissues. ACE2 exhibited the high expression level in the testis, small intestine, and thyroid (6.33 < average TPM < 46.53), a secondary level in the pancreas, lung, ovary, fallopian tube, breast, vagina, and minor salivary gland (1 < average TPM < 2.38), and the low level in blood, muscle, spleen, nerve, prostate, bladder, live, uterus, pituitary, and adrenal gland (0.019 < average TPM < 0.70). TMPRSS2 exhibited a high expression level in the prostate, stomach, lung, thyroid, small intestine, pancreas, liver, and minor salivary gland (12.72 < average TPM < 178.1), the secondary level in vagina, breast, fallopian tube, pituitary, and bladder (1.37 < average TPM < 6.73), and a low level in other tissues. The other six SARS-CoV-2-required genes especially ATP6AP1 and RAB7A showed a broad expression in all tissues (2.18 < average TPM < 312.3) (**Figure 3E**). These findings mean that the thyroid, small intestine, testis, and lung may show a higher SARS-CoV-2 infection risk relative to other tissues because both ACE2 and TMPRSS2 expression were higher.

To further explore whether different cancer types had different SARS-CoV-2 infection risks, we evaluated specific expression profiles of SARS-CoV-2-required genes in the tumor tissues. We observed that ACE2 and TMPRSS2 had a lower expression level across all cancer types, compared to other SARS-CoV-2-required genes (**Figure 3F**). Similar to the normal organ tissues, the corresponding tumor tissues also showed an obvious expression difference for both ACE2 and TMPRSS2, of which BLCA, CECS, CHOL, COAD, ESCA, KIRP, LUAD, LUSC, PAAD, READ, STAD, THCA, and UCEC had the higher expressions of ACE2 and TMPRSS2 compared with other tumor tissue types (**Figure 3G**). These results indicate that the above cancer types might have a higher SARS-CoV-2 infection risk.

### Identification of SARS-CoV-2-Required Gene Expression in Pan-Cancers

**Figure 4:**
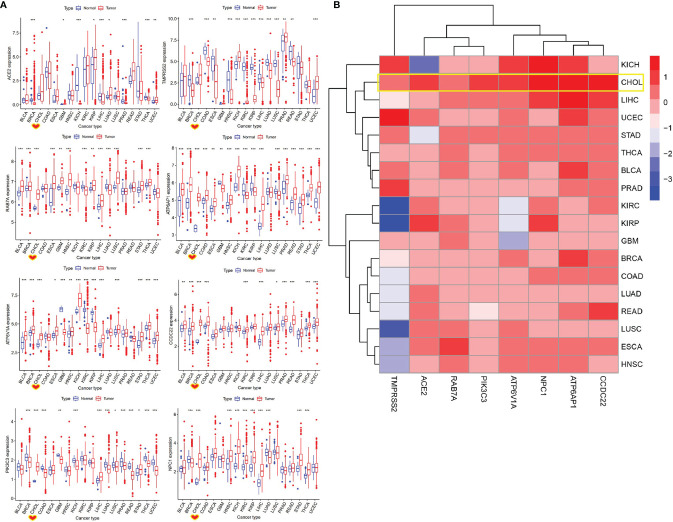
Identification of differentially expressed SARS-CoV-2-required genes between cancer and control samples. **(A)** The expression of SARS-CoV-2-required genes between cancer and control samples. *P < 0.05, **P < 0.01, ***P < 0.001. **(B)** Heatmap of log_2_ (fold change) for SARS-CoV-2-required genes between cancer and control samples. Blue represents downregulation, Red presents upregulation.

To evaluate whether cancer patients appear more vulnerable to SARS-CoV-2 relative to healthy individuals, we performed a differential expression analysis for SARS-CoV-2-required genes across 18 cancer types that had more than 5 normal samples. We found that these genes showed different expression levels between 18 types of tumor tissues and corresponding control tissues. ACE2 exhibited a significantly higher level in GBM, KIRP, LUAD, and UCEC, as well as an obviously lower level in BRCA, KICH, LIHC, PRAD, and THCA, compared with that in normal tissues (*P* < 0.05) (**Figure 4A** and **Table S4**). Notably, 75% of SARS-CoV-2-required genes including RAB7A, PIK3C3, ATP6AP1, ATP6V1A, CCDC22, and NPC1 showed a consistently significant upregulation in CHOL relative to normal tissues (1.09E-06 < *P* < 0.0001). In addition, ACE2 and TMPRSS2 appeared to be upregulated in CHOL compared to normal tissues (*P* > 0.05), indicating a possibly high risk of SARS-CoV-2 infection for CHOL (**Figures 4A, B** **)**. Furthermore, 62.5% of SARS-CoV-2-required genes containing ACE2, TMPRSS2, ATP6AP1, ATP6V1A, and CCDC22 exhibited a significant upregulation in UCEC relative to normal tissue, while only PIK3C3 showed an obvious downregulation in this cancer.

### SARS-CoV-2-Required Gene Expressions Affect Pan-Cancer Stage and Prognosis

To explore the role of SARS-CoV-2-required genes in pan-cancer prognosis, we performed the survival and univariate Cox proportional hazards regression analyses for these genes in all cancer types. Survival analysis indicated that ACE2 expression showed a good overall survival in KIRC, OV, and MESO. ATP6AP1 and ATP6V1A showed a positive association with good survival in PAAD and KIRC, respectively. The high CCDC22 or NPC1 expressions had a positive relationship with the poor survival in LIHC or MESO. RAB7A expression showed a poor prognosis in LIHC, UCEC, and PAAD, while exhibited a good prognosis in UVM (*P* < 0.01) (**Table S5**). Notably, univariate Cox proportional hazards regression analysis suggested that only CCDC22, RAB7A, ATP6V1A, and ATP6AP1 expressions were significantly associated with the overall survival of LAML and showed a high risk for poor prognosis. Moreover, our results also suggested that only NPC1, RAB7A, CCDC22, ATP6V1A, and ATP6AP1 expressions were obviously correlated with overall survival of LIHC and had a high risk for poor prognosis (HR >1, 6.78E-05 < *P* < 0.08) (**Figure S1A** and **Table S5**). While, only ACE2, PIK3C3, ATP6V1A, and ATP6AP1 expressions were obviously linked to the overall survival of KIRC and showed a low risk for poor prognosis (HR <1, 3.26E-09 < *P* < 0.009). These findings suggest a possibility that patients with LAML or LIHC infected after SARS-CoV-2 may have a poor prognosis.

Dai et al. (24) observed that patients with metastatic cancer (Stage IV) had a higher risk of death, ICU admission, and severe conditions, compared with no cancers or cancers without metastasis. In this study, we assessed the expression status of SARS-CoV-2-required genes in the different stage types of pan-cancers and predicted the potential risk of SARS-CoV-2 infection of cancer patients with high or low stage type. In the GSCA database, we found that multiple SARS-CoV-2-required genes were downregulated in the high stage type (Stage III or IV) of KIRC compared with low stage type (Stage I or II) (**Figure S1B** and **Table S6**). We then confirmed the results in GEPIA 2 database, and observed that ACE2, TMPRSS2, RAB7A, ATP6AP1, ATP6V1A, and PIK3C3 were significantly downregulated in high stage of KIRC compared with low stage (3.68 < F value < 10.4; 1.21E-06 < Pr (>F) < 0.012). NPC1 and CCDC22 also showed a decreased tendency in the high stage relative to the low stage. This means that patients with a low stage of KIRC may have a higher SARS-CoV-2 infection risk than those with a high stage.

### SARS-CoV-2-Required Gene Expressions Are Related to Immune Response and the Tumor Microenvironment in Pan-Cancers

The TME comprised of stromal cells, immune cells, fibroblasts, blood vessels, endothelial cell precursors, etc., plays an important role in the initiation and maintenance of tumorigenesis (25) and affects the resistance to chemotherapy and radiotherapy, metastasis, and recurrence of cancer patients (26). To understand the association of SARS-CoV-2-required gene with TME in pan-cancers, we performed a spearman correlation analysis according to the ESTIMATE immune, stromal, and estimate scores. RAB7A, PIK3C3, ATP6AP1, ATP6V1A, and NPC1 expressions were found to show a consistently positive relationship with immune, stromal, and estimate scores in DLBC. Similarly, RAB7A, ATP6V1A, CCDC22, and NPC1 expressions had a consistently positive association with immune, stromal, and estimate scores in LAML. While TMPRSS2, PIK3C3, ATP6AP1, and ATP6V1A expressions exhibited a consistently negative relationship with immune, stromal, and estimate scores in KICH (**Figure S2A** and **Table S7**). Moreover, we further explored their roles in modulating cancer stem cells by measuring RNAss and DNAss. As shown in **Figure S2B** and **Table S7**, SARS-CoV-2-required genes might be linked to cancer stem cells purity in cancers, especially DLBC, TGCT, and THYM. Overall, these findings indicate that these genes could involve TME. The high expression levels of RAB7A, PIK3C3, ATP6AP1, ATP6V1A, CCDC22, and NPC1 were significantly associated with lower tumor purity of patients with hematologic cancer LAML, while TMPRSS2, PIK3C3, ATP6AP1, and ATP6V1A expressions were obviously correlated with higher tumor purity of KICH patients.

Immune subtypes, containing wound healing (C1), INF-gamma dominant (C2), inflammatory (C3), lymphocyte depleted (C4), immunologically quiet (C5), and TGF-β dominant (C6) are closely linked to overall survival and progression-free interval of cancer patients. For cancer patients, the C3 immune subtype shows the best prognosis, while C2 and C1 exhibit poor outcomes. Patients with the C4 or C6 immune subtypes have the least favorable outcome (17). We then explored the correlation of SARS-CoV-2-required genes with immune response. All SARS-CoV-2-required genes were found to be involved in six immune infiltration types in human tumors (*P <* 0.001) (**Figure S2C** **)**. Moreover, in LUSC, high ACE2 and NPC1 expressions were found to be associated with decreased C3 immune infiltration and correlated with increased C1, C2, and C6 immune infiltration (**Figure S2D**). In contrast, upregulation of RAB7A, ATP6V1A, and PIK3C3 was linked to the increased C3 and the decreased C4 in KICH (**Figure S2E**). These findings indicate that by affecting immune subtypes, ACE2 and NPC1 expressions may associate with a less favorable outcome in LUSC, and RAB7A, ATP6V1A, and PIK3C3 expressions might link to a favorable outcome in KICH.

### SARS-CoV-2-Required Gene Expressions Correlate With Immune Cell Infiltration in Pan-Cancers

**Figure 5:**
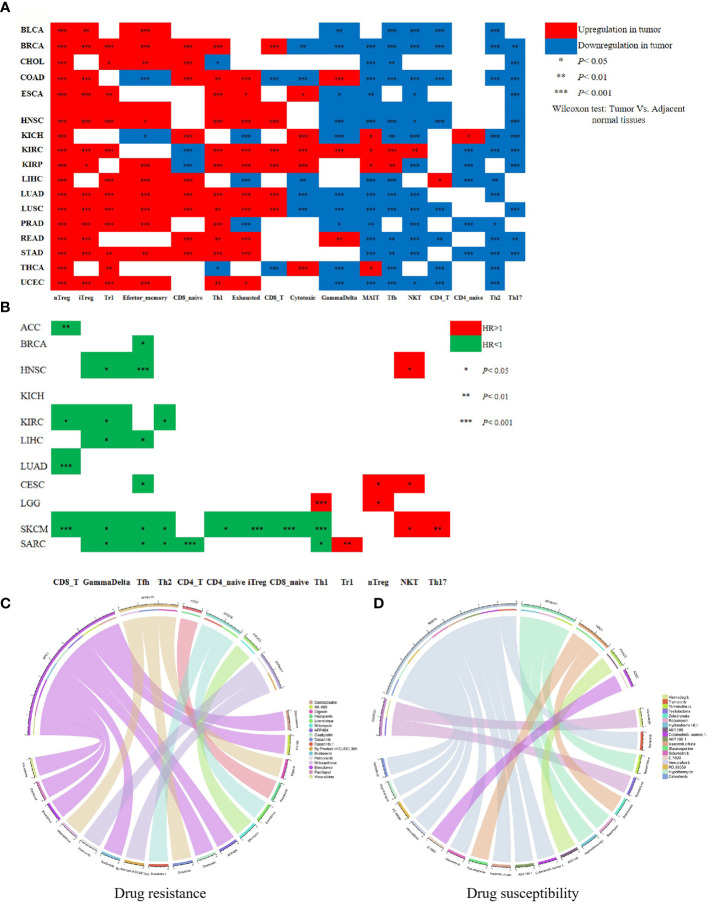
Immune cell infiltration involved in the prognosis of cancer patients and SARS-CoV-2-required genes associated with drug sensitivity. **(A)** The abundance of immune cell types between tumor and adjacent tissue in 17 cancers. **(B)** The relationship between immune cell types and the prognosis of cancer patients. **(C)** The relationship between drug sensitivity and SARS-CoV-2-required genes. **(D)** The positive relationship between drug sensitivity and SARS-CoV-2-required genes. *P < 0.05, **P < 0.01, ***P < 0.001.

Immune cell infiltration, including T cell (CD3+/CD8+/CD4+T, memory/effector T cell, and regulatory T cell), T helper 1 (TH1) cell, T helper 17 (TH17) cell, T helper 2 (TH2) cell, natural killer (NK) cell, plays a crucial role in inhibiting tumor cells or providing supports for tumor growth, and associates with a prognosis of 17 human cancers (24, 27–29). We utilized the ImmuCellAI database to perform a differential level analysis for immune cell infiltration between tumor and adjacent normal tissues and conducted a comprehensive analysis for the prognostic value of the major immune cell types across pan-cancers. We observed a broadly different abundance of immune cell types between tumor and adjacent tissues in 17 cancers. As shown in **Figure 5A**, nTreg, iTreg, Tr1, and Th1 were obviously enriched in the nidus of most cancer types. Conversely, several antitumor cells containing NKT, Th2, and Th17 exhibited a lower infiltration in most tumor types than the corresponding adjacent tissues. Especially, most immune cell types such as nTreg, iTreg, Tr1, CD8 naive, Th1, Exhausted, CD8+T, Cytotoxic, GammaDelta, MAIT, Tfh, NKT, CD4+T, Th2, and Th17 were enriched in the nidus or adjacent tissues of LUAD and LUSC. In addition, we found that CD8+T, GammaDelta, and Tfh were correlated with favorable prognoses in most cancers analyzed. Conversely, nTreg, NKT, and TH17 were indicative of poor prognosis, which is consistent with another previous report (24). Notably, CD8+T showed a strong correlation with the good prognosis of LUAD patients (**Figure 5B** **)**. Moreover, eight SARS-CoV-2-required genes had a positive or negative association with the abundance of most immune cell types in different cancer types (FDR < 0.01) (**Table S8**), among which, the expressions of ACE2 and NPC1 were negatively associated with the abundance of CD8+T (**Table S8**). These results suggest a relationship between SARS-CoV-2-required genes and immune cell infiltration and prognosis in pan-cancers. SARS-CoV-2-induced ACE2 and NPC1 elevation may have a negative influence in CD8+T of LUAD patients, which may result in a poor prognosis.

### SARS-CoV-2-Required Genes Affect Anti-Cancer Drug Sensitivity

Anti-cancer drug resistance is implicated in the therapeutic effect and prognosis of cancer patients. In this study, we evaluated the influence of SARS-CoV-2-required genes in anti-cancer drug sensitivity. As summarized in **Table S9**, SARS-CoV-2-required genes showed a broad influence on anti-cancer drug sensitivity. ACE2, RAB7A, PIK3C3, ATP6AP1, NPC1, and ATP6V1A had a negative association with the sensitivity of 16 anti-cancer drugs (*P* < 0.05). Especially, ACE2 expression was significantly associated with the decreased sensitivity of Pazopanib (advanced renal cell cancer and soft tissue sarcoma). NPC1 expression showed an obviously negative relationship with the sensitivity of Dexrazoxane (a cardioprotective agent against the cardiotoxic side effects of chemotherapeutic drugs), Oxaliplatin (carcinoma of the colon or rectum), Ifosfamide (testicular, ovarian, cervical, and bladder cancers, osteocarcinoma, small cell lung cancer, and non-Hodgkin’s lymphoma), Elesclomol (metastatic melanoma), Paclitaxel (Kaposi’s sarcoma and cancer of the lung, ovarian, and breast), and Vinorelbine (metastatic non-small cell lung carcinoma) (*P* < 0.01) (**Figure 5C**), suggesting that the upregulation of ACE2 and NPC1 may reduce the curative effect of these drugs. ACE2, RAB7A, CCDC22, ATP6AP1, NPC1, and PIK3C3 exhibited a positive relationship with sensitivity of 18 anti-cancer drugs (*P* < 0.01) (**Figure 5D**).

### Correlation Between OAS1, ACE2, TMPRSS2, and Lung Cancer-Related Genes and Their Influence on the Prognosis of Lung Cancer

**Figure 6:**
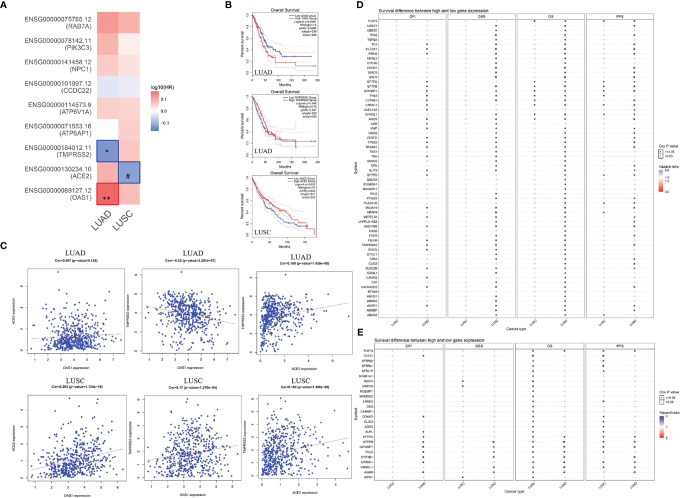
Correlation between OAS1, ACE2, and TMPRSS2, and effect of lung cancer-related genes in the prognosis of lung cancer. **(A, B)** Survival analysis for SARS-CoV-2-required/susceptibility genes in LUAD and LUSC. **(C)** Correlation between OAS1, ACE2, and TMPRSS2. **(D)** Association of lung cancer-related genes with the prognosis of LUAD. **(E)** Association of lung cancer-related genes with the prognosis of LUSC. *P < 0.05; **P < 0.01; ^#^0.05 < *P* < 0.1.

In this study, we found that several SARS-CoV-2-related genes may affect the prognosis of lung cancer patients. To further explore the potential mechanism of these genes on the prognosis of lung cancer, we performed a correlation and survival analysis for SARS-CoV-2-related genes and lung cancer-related genes in lung cancer. We found that OAS1, ACE2, TMPRSS2 were associated with overall survival of lung cancer (**Figure 6A**). High OAS1 was associated with the poor overall survival of LUAD, while high TMPRSS2 expression showed a good overall survival in LUAD. ACE2 expression had a positive association with overall survival of LUSC (**Figure 6B**). Correlation analysis indicated that OAS1 expression had a negative correlation with TMPRSS2 expression in LUAD. ACE2 expression showed a positive association with TMPRSS2 expression in LUAD. OAS1 expression exhibited no relationship with ACE2 expression in LUAD. For LUSC, there was a positive association between OAS1, TMPRSS2, and ACE2 expressions (**Figure 6C**). Differential gene expression analysis identified 268 downregulated and 69 upregulated differentially expressed genes (DEGs) in LUAD as well as 561 downregulated and 296 upregulated DEGs in LUSC. Among these DEGs, 35 upregulated and 236 downregulated genes showed a consistent association with both LUAD and LUSC (**Table S10**). We then estimated the association of 271 DEGs with OAS1, TMPRSS2, and ACE2 expressions in LUAD and LUSC. We found that ACE2 expression had a positive correlation with GPX2 expression and a negative correlation with SLC2A1 expression in both LUAD and LUSC. OAS1 expression was positively related to C1QB expression in both LUAD and LUSC. TMPRSS2 expression was negatively associated with 13 lung cancer-related genes and showed a positive correlation with 130 lung cancer-related genes (**Table S11**). Finally, we carried out a survival analysis for the above 146 lung cancer-related genes in LUAD and LUSC. The results showed that 64 lung cancer-related genes were linked to overall survival of LUAD patients, and 26 lung cancer-related genes were correlated with overall survival of LUSC patients. Among these genes, high SLC2A1 expression was associated with the poor overall survival of LUAD patients, but not in LUSC. Notably, FLRT3, CYP4B1, CHRDL1, SFTPC, SFTPB, and AGER, which had a positive correlation with TMPRSS2, were significantly downregulated in LUAD and LUSC. LUAD and LUSC patients with high FLRT3 expression had poor overall survival. CYP4B1, CHRDL1, SFTPC, SFTPB, and AGER expressions were linked to the good overall survival in LUAD patients (**Figures 6D, E**, and **Table S11**), whereas the opposite was true in patients with LUSC, indicating that these genes may have different effects on prognosis of LUAD and LUSC.

### Upstream Regulators of SARS-CoV-2-Required Genes

**Figure 7:**
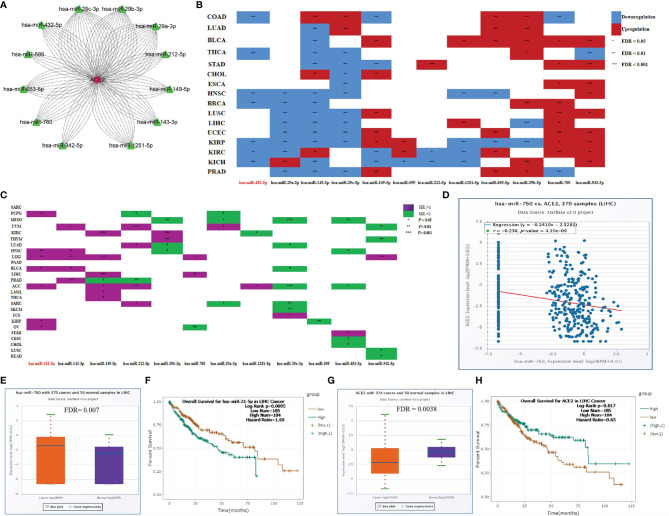
Upstream regulators of ACE2. **(A)** microRNAs targeting ACE2. **(B)** Differentially expressed microRNAs between cancer tissues and control tissues. **(C)** Association between microRNAs and the prognosis of cancers. **(D)** Correlation between miR-760 and ACE2 in LIHC. **(E)** Expression difference of miR-760 between LIHC tissues and control tissues. **(F)** Association between miR-760 and the prognosis of LIHC. **(G)** Expression difference of ACE2 between LIHC tissues and control tissues. **(H)** Association between ACE2 and the prognosis of LIHC. *P < 0.05; **P < 0.01; ***P < 0.1.

Non-coding RNA is widely acknowledged to regulate the expression of target genes. To identify the upstream regulators of SARS-CoV-2-required genes and explore the potential treatment and intervention targets for SARS-CoV-2, we used the starBase database to predict microRNAs targeting SARS-CoV-2-required genes. As summarized in **Table S12**, one hundred and forty-six microRNAs were found to be upstream regulators of SARS-CoV-2-required genes. In this study, we focused on the ACE2 and its upstream microRNAs. Total 12 microRNAs containing miR-29a-3p, miR-29b-3p, miR-143-3p, miR-149-5p, miR-29c-3p, miR-432-5p, miR-599, miR-653-5p, miR-760, miR-942-5p, miR-1251-5p, and miR-212-5p were predicted to target ACE2 and showed a significantly negative or positive correlation with ACE2 expression in 30 cancer types (-0.42 < Cor < 0.46; *P* < 0.05) (**Figure 7A**). These microRNAs also exhibited significantly differential expression between 17 types of cancer samples and the corresponding control samples (**Figure 7B**) and had a different effect on the prognosis of 25 cancer types (**Figure 7C**). In particular, miR-760, targeted ACE2, had a negative relationship with ACE2 expression (Cor = -0.24; *P* = 4.55E-06) (**Figure 7D**), and was markedly upregulated in LIHC (FDR= 0.007) (**Figure 7E**). Its upregulation was positively linked to the poor prognosis of LIHC patients (HR=1.78; *P* = 0.0015) (**Figure 7F**). Conversely, ACE2 was downregulated in LIHC (FDR = 0.0038) (**Figure 7G**), and its upregulation showed a positive association with the favorable prognosis of LIHC patients (HR = 0.65; *P* = 0.017) (**Figure 7H**). These findings indicate that microRNAs could be the potential regulator of SARS-CoV-2-required genes. Notably, miR-760 may have the potential to serve as a treatment and intervention target for SARS-CoV-2 because of its inhibitory effect on ACE2.

## Discussion

Four epidemiological investigations revealed that cancer patients appear more vulnerable to SARS-CoV-2 and show poor outcomes compared with non-cancer patients (10–13). Moreover, several research teams have demonstrated that host-specific genetic factors play an important role in SARS-CoV-2 susceptibility and COVID-19 outcomes (5–9). In this study, we aimed to explore whether SARS-CoV-2-required genes and host genes and variants play a critical role in the SARS-CoV-2 susceptibility of cancer patients and poor COVID-19 outcomes of cancer patients infected with SARS-CoV-2.

Firstly, we evaluated the response of 14 host genes related to SARS-CoV-2 susceptibility and COVID-19 outcomes in multiple cell types of the respiratory system after SARS-CoV-2 infection. We found that SARS-CoV-2 can significantly elevate OAS1 and PLEKHA4 expressions in turbinate, lung tissues, and NHBEs. OAS1 showed a broad expression in different human organs and tumor tissues of the HPA database, with low organ and cancer specificity. Zhou et al. identified that increased plasma OAS1 protein level is positively associated with reduced COVID-19 susceptibility and poor outcomes in 14,134 cases and 1.2 million controls. Collectively, these findings suggested that OAS1 may serve as a protective factor against SARS-CoV-2 infection and poor COVID-19 outcomes in the wide organs and tissues. We further explored the effect of SNPs located on these 14 host genes on expressions of potential key genes *via* expression quantitative trait locus (cis-eQTL) analysis in 33 cancer types. Rs4801778-T (PLEKHA4), rs11919389-C (ZBTB11), rs13050728-C (IFNAR2), and rs10774671-A (OAS1) exhibited a positive or negative regulation in TULP2, HSD17B14, LOC285359, LOC100009676, SENP7, IFNAR2, OAS3, and OAS1 in multiple cancer types. Especially, rs10774671-A (OAS1) showed a consistent negative association with OAS1 expression in BLCA, HNSC, KIRC, KIRP, LGG, PCPG, PRAD, TGCT, and THCA. Taking together, these findings indicate that BLCA, HNSC, KIRC, KIRP, LGG, PCPG, PRAD, TGCT, and THCA patients carrying rs10774671-A (OAS1) genotype may be more likely to have poor COVID-19 outcomes relative to those carrying rs10774671-G because individuals carrying rs10774671-A will have the lower expression of OAS1, which serves as a protective factor against SARS-CoV-2 progress and poor COVID-19 progress outcomes.

Subsequently, we assessed the response of eight SARS-CoV-2-required genes in multiple cell types of the respiratory system after SARS-CoV-2 infection. We observed that SARS-CoV-2 increased ACE2 and NPC1 expression in normal/tumor tissues or cells of the human respiratory system, similar to one previous report (4). We then evaluated expression profiles of SARS-CoV-2-required genes in human normal and pan-cancer tissues. We found that ACE2 and TMPRSS2 showed an obvious expression difference between different human tissues, while other SARS-CoV-2-required genes had a widely high or medium expression level in all tissues. Compared with other tissues, the small intestine, and thyroid, testis, lung, pancreas, breast, and fallopian had higher expression levels of SARS-CoV-2-required genes. For tumor tissues corresponding to the above organs, BLCA, CECS, CHOL, COAD, ESCA, KIRP, LUAD, LUSC, PAAD, READ, STAD, THCA, and UCEC showed higher expressions of ACE2 and TMPRSS2 compared with other tumor tissue types. These results may mean a higher SARS-CoV-2 susceptibility in these tissues and the corresponding tumor tissues.

We further analyzed the expression profiles for eight SARS-CoV-2-required genes in pan-cancers. Seventy-five percent of SARS-CoV-2-required genes including RAB7A, PIK3C3, ATP6AP1, ATP6V1A, CCDC22, and NPC1 were found to show a consistently significant upregulation in CHOL relative to normal tissues. In addition, ACE2 and TMPRSS2 appeared to be upregulated in CHOL compared to normal tissues. These results indicate that CHOL patients potentially have a higher risk of SARS-CoV-2 infection compared with healthy subjects. In addition, we observed an association of SARS-CoV-2-required genes with the poor or good prognosis of multiple cancer types by survival analysis and univariate Cox proportional hazards regression analysis. Among which, CCDC22, RAB7A, ATP6V1A, and ATP6AP1 expressions were significantly associated with the poor overall survival of LAML, suggesting a possibility that patients with hematological cancer (LAML) after SARS-CoV-2 infection may have a poor prognosis, and might support conclusions by Bernard et al. (1) and Dai et al. (11) that SARS-CoV-2-infected patients with hematological cancer have the highest frequency of severe events including death rates and ICU admission. By GSCA and GEPIA 2 databases, we confirmed that ACE2, TMPRSS2, RAB7A, ATP6AP1, ATP6V1A, and PIK3C3 were significantly downregulated in the high stage of KIRC compared with the low stage. NPC1 and CCDC22 also showed a reduced tendency in the high stage. These results mean that patients with a low stage of KIRC may have a higher SARS-CoV-2 infection risk than those with a high stage.

We analyzed the relationship of SARS-CoV-2-required genes with TME and immune response in pan-cancers and found that their expression was significantly associated with tumor purity of patients with LAML and KICH. Immune subtypes were reported to involve overall survival and progression free intervals of cancers. C3 (inflammatory) shows the best prognosis in cancer patients, while C2 (IFN-γ dominant), C1 (wound healing), C4 (lymphocyte depleted), and C6 (TGF-β dominant) exhibit the poor outcome (7). In our analysis, the high ACE2 and NPC1 expressions were found to be associated with the decreased C3 immune infiltration of LUSC, and correlate with increased C1, C2, and C6 immune infiltration. Immune cell infiltration plays a crucial role in the prognosis of multiple human cancers (24, 27–29). Similar to the previous report, we found that CD8+T, GammaDelta, and Tfh were correlated with the favorable prognosis in most of the cancer types analyzed; while nTreg cells, NKT, and TH17 cells were indicative of poor prognosis (24), which may be affected by SARS-CoV-2-required genes. Notably, CD8+T showed a positive correlation with the good prognosis of LUAD patients, and the expressions of ACE2 and NPC1 were negatively associated with the abundance of CD8+T. Given that ACE2 and NPC1 were significantly upregulated in the normal tissues and cells or tumor cells of the respiratory system infected after SARS-CoV-2, these findings indicate that LUSC or LUAD patients infected with SARS-CoV-2 may have a worse outcome because SARS-CoV-2-induced ACE2 and NPC1 elevation may have a negative influence in C3 and a positive effect on the C1, C2, and C6 immune infiltration of LUSC, or have a negative influence in CD8+T of LUAD. This also may support the conclusions of Bernard et al. (10) and Dai et al. (11) that patients with lung cancer have a high frequency of severe events.

To further explore the potential mechanism of SARS-CoV-2-related genes on the prognosis of lung cancer, we performed a correlation and survival analysis for these genes and lung cancer-related genes in lung cancer. Our results suggested that OAS1, ACE2, and TMPRSS2 expressions showed a different interaction in LUAD and LUSC and had a different effect on the prognosis of LUAD and LUSC. Notably, OAS1 expression showed a negative association with TMPRSS2 expression in LUAD, while exhibited a positive correlation with TMPRSS2 expression in LUSC, indicating that upregulation of OAS1 may decrease TMPRSS2 expression in LUAD but may increase TMPRSS2 expression in LUSC. We also found that these genes showed a consistent association with 90 lung cancer-related genes having different influences on the prognosis of LUAD or LUSC patients. Especially, five lung cancer-related genes including CYP4B1, CHRDL1, SFTPC, SFTPB, and AGER were consistently downregulated in both LUAD and LUSC had a positive correlation with TMPRSS2, exhibited an opposite effect on the prognosis of LUAD and LUSC. These findings indicate that LUSC and LUAD patients may have a varying degree of adverse outcomes if they are infected with SARS-CoV-2 because of the opposite interaction between OAS1 and TMPRSS2 in LUAD and LUSC as well as the opposite effect of these lung cancer-related genes on the prognosis of LUAD and LUSC.

We evaluated the influence of SARS-CoV-2-required genes in anti-cancer drug sensitivity, a common event influencing the therapeutic effect and prognosis of cancer patients. SARS-CoV-2-required genes were found to show a broad influence in anti-cancer drug sensitivity. Notably, ACE2 and NPC1, elevated in human cells or tissues infected with SARS-CoV-2, were found to be significantly associated with the decreased drug sensitivity (Pazopanib, Dexrazoxane, Oxaliplatin, Ifosfamide, Elesclomol, Paclitaxel, and Vinorelbine) of multiple cancer types including small cell lung cancer and metastatic non-small cell lung carcinoma, suggesting that patients with cancers (especially lung cancers) after SARS-CoV-2 infection may have a poor outcome because of the negative effect of SARS-CoV-2-induced upregulation of ACE2 and NPC1 on these anti-cancer drug sensitivity.

MicroRNA is dysregulated in various cancers *via* different mechanisms, which in return influences cancer hallmarks such as tumor cell proliferation, death inhibition, metastasis, and angiogenesis (30). In the current analysis, 146 microRNAs were found to be the upstream regulators of SARS-CoV-2-required genes. Total 12 microRNAs were predicted to target ACE2, with a significantly negative or positive correlation with ACE2 expression in 30 cancer types. Especially, miR-760, a broadly downgraded tumor suppressor in various cancer types (30–33), may have the potential to serve as a treatment and intervention target for SARS-CoV-2 because of its inhibitory effect on ACE2. Elevating miR-760 could be beneficial for cancer treatment and SARS-CoV-2 prevention.

**Figure 8:**
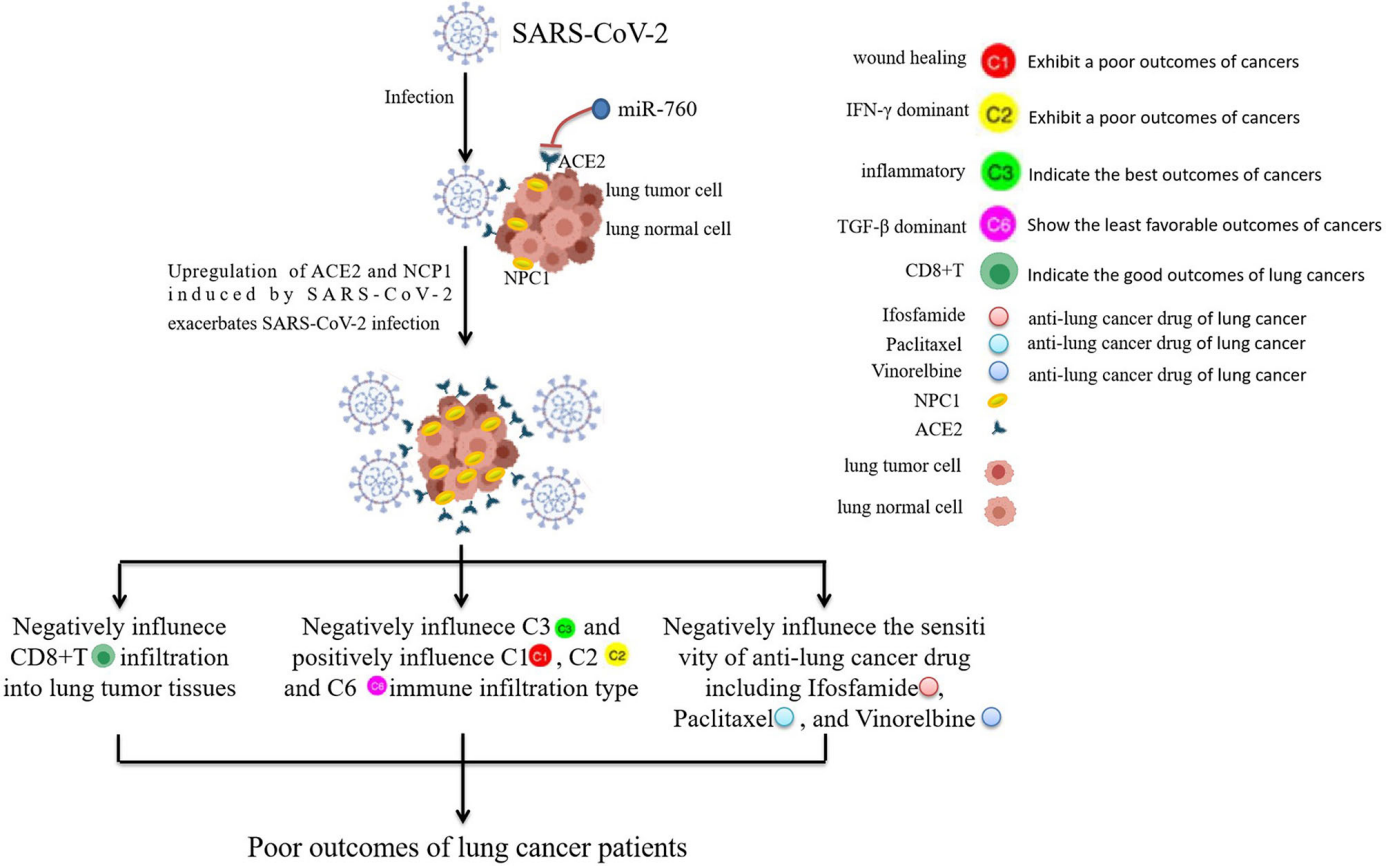
The prediction of the worse outcome for lung cancer patients infected with SARS-CoV-2. Lung cancer patients infected with SARS-CoV-2 may have a worse outcome because SARS-CoV-2-induced ACE2 and NPC1 elevation may have a negative influence on the immune response of LUSC and CD8+T infiltration of LUAD, and negatively affect the sensitivity of anti-cancer drugs including Ifosfamide, Paclitaxel, and Vinorelbine. miR-760 may have the potential to serve as a treatment and intervention target for SARS-CoV-2 because of its targeted inhibition effect on ACE2.

In conclusion, the findings in this study demonstrate that BLCA, HNSC, KIRC, KIRP, LGG, PCPG, PRAD, TGCT, and THCA patients carrying rs10774671-A (OAS1) genotype may have a higher risk for poor COVID-19 outcomes relative to those who carry rs10774671-G. SARS-CoV-2-required genes were correlated with TME, immune response, and infiltration, overall survival, anti-cancer drug sensitivity of pan-cancers. CHOL patients may have a higher risk of SARS-CoV-2 infection than healthy subjects. As shown in **Figure 8**, lung cancer patients infected with SARS-CoV-2 may have a worse outcome because SARS-CoV-2-induced ACE2 and NPC1 elevation, which in turn promotes further SARS-CoV-2 invasion, may influence the immune subtypes of LUSC and immune infiltration in CD8+T of LUAD, and affect the sensitivity of anti-cancer drug. LUSC and LUAD patients may have a varying degree of adverse outcomes if they are infected with SARS-CoV-2. OAS1, ACE2, and miR-760 could serve as treatment and intervention targets for SARS-CoV-2. Future studies are needed to confirm the results by *in vitro* and *in vivo* experiments.

## Data Availability Statement

The data generated in this study are publicly available in the TCGA, GEO (GSE163959 and GSE147507), GTEx, HPA, GSCA, GEPIA 2, PancanQTL, starbase 3.0, CellMiner, and ImmuCellAI databases.

## Author Contributions

LX and XH designed research. XH, HL and HZ drafted the manuscript and revised the paper. XH, HZ, and HL performed analyses. XH, TW, LP, LT, QZ, XG, WL, AC, QD, YZ, HW, MH, DD, and ZL participated in data consolidation and plotting. All authors contributed to the article and approved the submitted version.

## Funding

This work was supported by the key discipline construction Program of Shanghai (no. ZK2019B13).

## Conflict of Interest

The authors declare that the research was conducted in the absence of any commercial or financial relationships that could be construed as a potential conflict of interest.

## Publisher’s Note

All claims expressed in this article are solely those of the authors and do not necessarily represent those of their affiliated organizations, or those of the publisher, the editors and the reviewers. Any product that may be evaluated in this article, or claim that may be made by its manufacturer, is not guaranteed or endorsed by the publisher.

## Supplementary Material

Supplementary Figure 1 The prognosis analysis for SARS-CoV-2-required genes in 33 cancer types and the different assessment of SARS-CoV-2-required genes in different stages of pan-cancers. **(A)** Univariate Cox regression analysis for SARS-CoV-2-required genes. **(B)** SARS-CoV-2-required genes ACE2, TMPRSS2, RAB7A, ATP6AP1, ATP6V1A, and PIK3C3 were downregulated in high stage type of KIRC compared with low stage type identified by GSCA (http://bioinfo.life.hust.edu.cn/GSCA/#/) and GEPIA 2 (http://gepia2.cancer-pku.cn/#index) databases.

Supplementary Figure 2 SARS-CoV-2-required genes associated with immune subtypes and tumor microenvironment. **(A)** Association of SARS-CoV-2-required gene expression with the ESTIMATE immune, stromal, and estimate scores. **(B)** Association of SARS-CoV-2-required gene expression with RNAss and DNAss. Association of SARS-CoV-2-required genes with immune subtypes in all cancer patients **(C)**, LUSC patients **(D)**, and KICH patients **(E)**.

Supplementary Table 7 SARS-CoV-2-required genes associated with immune subtypes and tumor microenvironment.

Supplementary Table 9 SARS-CoV-2-required genes associated with drug sensitivity.

Supplementary Table 10 Identification of differentially expressed genes in LUAD and LUSC, and their association with prognosis of lung cancer patients.

Supplementary Table 11 Correlation between OAS1, ACE2, TMPRSS2, and lung cancer-related genes.

Supplementary Table 12 Upstream regulators of SARS-CoV-2-required genes.
