# Supplementary figures and images for: The Potential Mechanism of Cancer Patients Appearing More Vulnerable to SARS-CoV-2 and Poor Outcomes: A Pan-Cancer Bioinformatics Analysis
Source: Front Immunol. 2022 Jan 10;12:804387. doi: 10.3389/fimmu.2021.804387 (PMC8784815; doi:10.3389/fimmu.2021.804387)

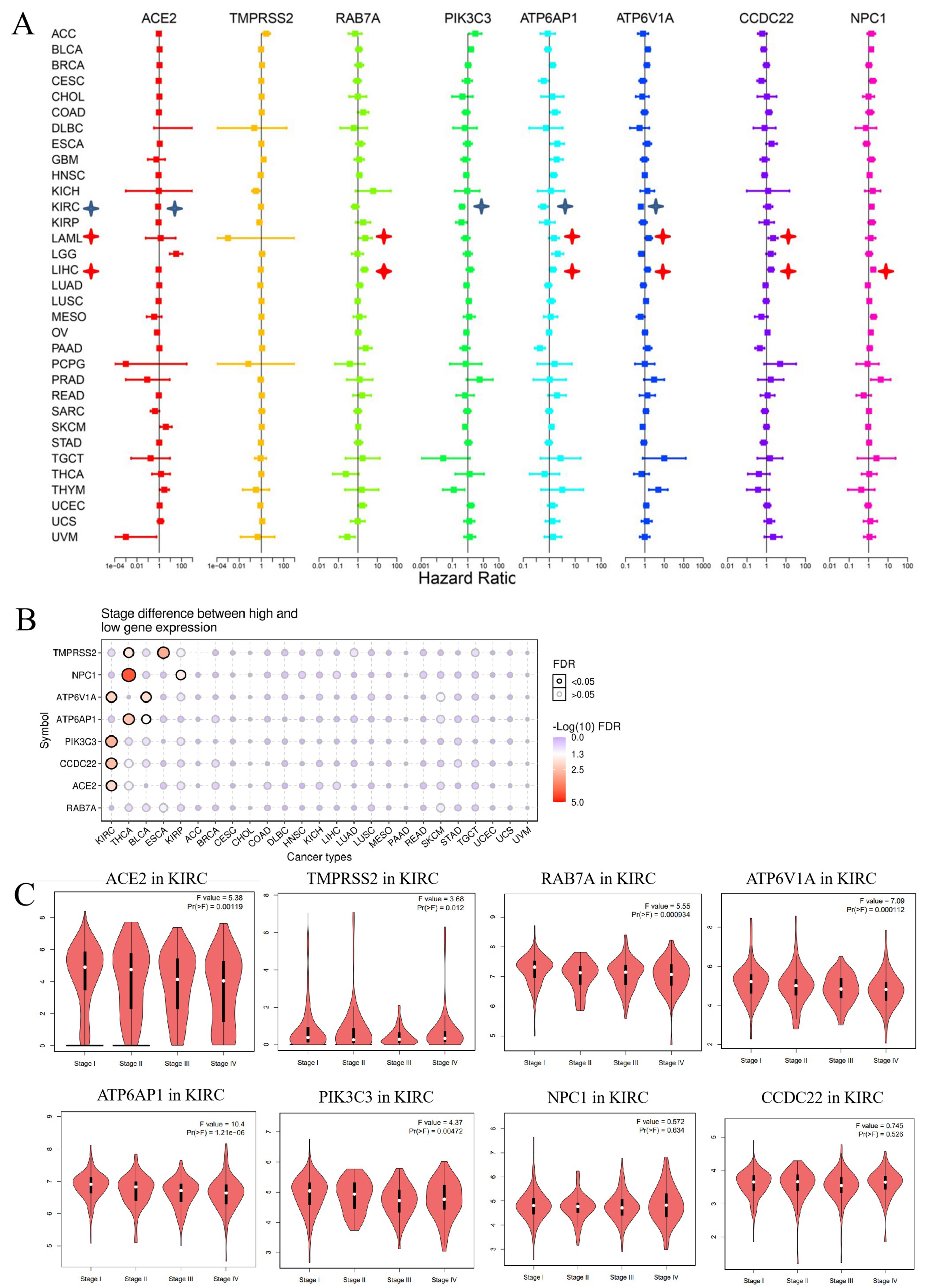

Supplement: Supplementary Figure 1 — The prognosis analysis for SARS-CoV-2-required genes in 33 cancer types and the different assessment of SARS-CoV-2-required genes in different stages of pan-cancers. (A) Univariate Cox regression analysis for SARS-CoV-2-required genes. (B) SARS-CoV-2-required genes ACE2, TMPRSS2, RAB7A, ATP6AP1, ATP6V1A, and PIK3C3 were downregulated in high stage type of KIRC compared with low stage type identified by GSCA (http://bioinfo.life.hust.edu.cn/GSCA/#/) and GEPIA 2 (http://gepia2.cancer-pku.cn/#index) databases. [file Image_1.jpeg]

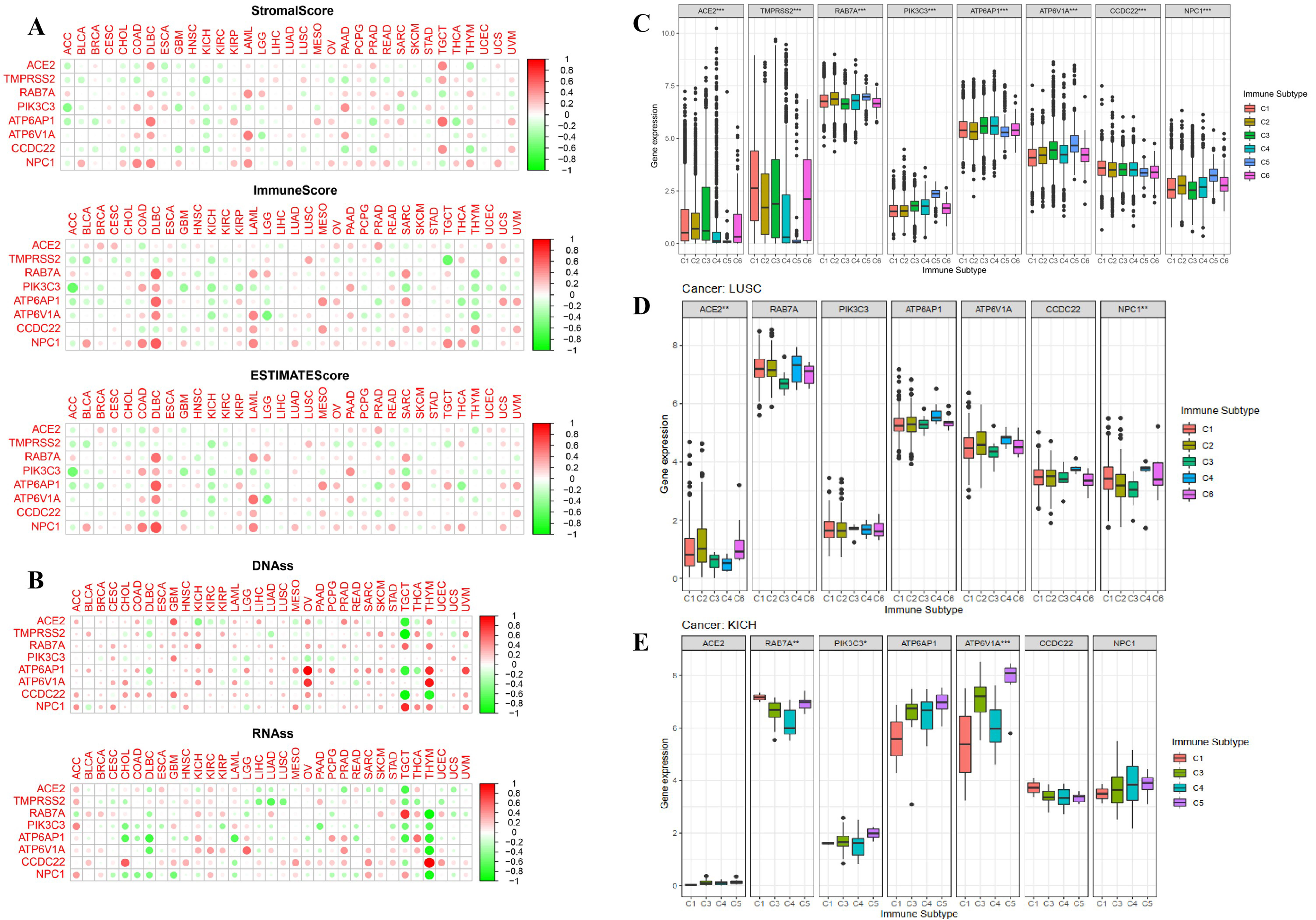

Supplement: Supplementary Figure 2 — SARS-CoV-2-required genes associated with immune subtypes and tumor microenvironment. (A) Association of SARS-CoV-2-required gene expression with the ESTIMATE immune, stromal, and estimate scores. (B) Association of SARS-CoV-2-required gene expression with RNAss and DNAss. Association of SARS-CoV-2-required genes with immune subtypes in all cancer patients (C), LUSC patients (D), and KICH patients (E). [file Image_2.jpeg]
